# 

**Published:** 1882-04

**Authors:** 


### COMMUNICATIONS.

The Dental Pulp—Its Pathology and Treatment........................ 155
The Essential Oils, and Eucalyptus Globulus in Dentistry.............. 165
Definite Causes of Caries...... ................................................ 175
Address to Graduating College.................................................. 179
Founding of the Ohio College of Dental Surgery............................ 186
Proceedings Mississippi Valley Dental Association......................... 187

### CORRESPONDENCE.

Dental Societies.......................................................................... 201

### EDITORIAL.

Illinois Dental Society.................................................................. 202
Kansas State Dental Association................................................... 202
Another Resolution........................................................................ 203
Home Again.................................................................................. 204
The Graduates.............................................................................. 204

### SELECTIONS.

Extraction of Teeth in Pregnant Women........................................ 205

